# Evidence of histone modification affecting *ARID1A* expression in colorectal cancer cell lines 

**Published:** 2022

**Authors:** Mehran Erfani, Mozhdeh Zamani, Pooneh Mokarram

**Affiliations:** 1 *Department of Biochemistry, Faculty of Medicine, Shiraz University of Medical Sciences, Shiraz, Iran*; 2 *Department of Biochemistry, School of Medicine, Hormozgan University of Medical Sciences, Bandar Abbas, Iran*; 3 *Autophagy Research Center, Shiraz University of Medical Sciences, Shiraz, Iran*

**Keywords:** ARID1A, Histone acetylation, Colorectal cancer, Epigenetics

## Abstract

**Aim:**

The current study aimed to focus on the role of histone deacetylation in reduced *ARID1A* expression in colorectal cancer cell lines.

**Background:**

ARID1A, a subunit of the switch/sucrose nonfermentable chromatin remodeling complex, has emerged as a bona fide tumor suppressor and is frequently downregulated and inactivated in multiple human cancers. Epigenetic modifications play an important role in dysregulation of gene expression in cancer. DNA methylation has been reported as an important regulator of *ARID1A* expression in colorectal cancer cell lines; however, the histone modification role in *ARID1A* suppression in colorectal cancer remains unclear.

**Methods:**

The expression levels of ARID1A mRNA were determined using real-time quantitative PCR in colorectal cancer cell lines including HCT116, SW48, HT29, SW742, LS180, and SW480. To evaluate the effect of histone deacetylation on ARID1A expression, all cell lines were treated with trichostatin A (TSA), a histone deacetylase inhibitor. SPSS software (Version 23) and GraphPad Prism (Version 6.01) were applied for data analysis using one-way ANOVA, followed by Tukey’s multiple comparison tests.

**Results:**

Treatment of colorectal cancer cell lines with TSA increased *ARID1A *expression in a cell line-dependent manner, suggesting that histone deacetylation is at least one factor contributing to *ARID1A* downregulation in colorectal cancer.

**Conclusion:**

Histone deacetylase inhibitors might provide a strategy to restore *ARID1A* expression and may bring benefits to the colorectal cancer patients with a broader range of genetic backgrounds.

## Introduction

 The molecular and genomic understanding of colorectal cancer (CRC) has progressed considerable in the last decade; however, CRC is still one of the most common causes of cancer deaths in the world (1, 2). CRC is a genetically heterogeneous disease and should thus be treated in a personalized way (3, 4). An increased understanding of the genetic and epigenetic mechanisms underlying the pathogenesis of CRC is crucial for personalized therapeutic strategies. 

The third most significantly mutated gene in human CRC is the adenine-thymine-rich interactive domain 1A (*ARID1A*) encoding gene (5), which is located on chromosome 1p36.11 and is a principal subunit of the SWI–SNF complex (switch/sucrose non-fermentable) (6, 7). The SWI/SNF complex is a chromatin remodeling complex utilizing the energy of ATP hydrolysis to regulate the transcription of certain genes by altering the chromatin structure around those genes (7). Numerous studies have identified *ARID1A* as a bona fide tumor suppressor during the oncogenic process, and its downregulation and mutations have been frequently reported in a broad spectrum of cancers (8), including gastrointestinal tract tumors (9-11). Notably, loss of *ARID1A* expression is associated with high risk or poor outcome of various cancers (6, 11-15). 

Data from The Cancer Genome Atlas project (TCGA) revealed that *ARID1A* is significantly mutated in CRC, with the highest frequency of mutations (~39%) in cancers of the MSI type (16-18). Analysis of the whole-exome sequencing revealed that these mutations often lead to a loss of *ARID1A* expression in tumors (17, 18). The tumor suppressive role of ARID1A was well-established in colorectal cancer. A recent study showed that the sporadic deletion of *ARID1A* in mice led to the spontaneous formation of invasive adenocarcinomas in the colon (5). In addition, Kishida et al. revealed that *ARID1A* expression loss was correlated with lymphatic invasion and early onset in T1 CRC (19). Moreover, knockdown of *ARID1A* significantly enhanced the migration activity of HCT116 human colon cancer cells (20).

Downregulation of the *ARID1A* gene in cancers is attributed to genomic deletion, DNA mutation, DNA methylation, and microRNA-mediated inhibition (8, 21-24). Although *ARID1A* is the frequent target of inactivating mutations in CRC (8, 16, 17), the available data indicates that mutation is only a component of the observed *ARID1A* gene inactivity in CRC. It is very likely that *ARID1A* gene expression is influenced by epigenetic modifications, like histone deacetylation and gene promoter CpG islands hypermethylation, which play important roles in carcinogenesis associated with transcriptional repression of genes regulating cell replication, DNA repair, tumor suppression, and apoptosis (25). The acetylation of histones is regulated by histone acetyltransferase (HAT) and histone deacetylase (HDAC). HDAC is able to remove the acetyl group from the histone lysine residue, increase the DNA-binding ability of histones, and make the promoter less accessible to transcriptional regulatory elements, finally causing transcriptional repression. The effect of HAT is, however, just the opposite. Together, they synergistically maintain the normal acetylation level of histone (26). Trichostatin A (TSA), a powerful and specific Class I and II histone deacetylase inhibitor (HDACi), is widely used to increase the expression of genes silenced by chromatin condensation. Moreover, TSA inhibits tumor growth and induces apoptosis in cancer cells, which indicates the potential application of this drug in epigenetic therapy against cancer (26, 27). Pharmacological inhibitors of class I and II HDAC activity are potent inducers of growth arrest, differentiation, and apoptosis of colon cancer cells in vitro and in vivo, indicating a role for these HDACs in tumor promotion (26, 28, 29).

Relatively few studies have been conducted investigating the importance of epigenetic modifications in the downregulation of *ARID1A* in cancer. Zhang *et al*. showed that *ARID1A* promoter hypermethylation and histone modification led to low mRNA expression of the *ARID1A* gene in breast cancer (21). We also previously suggested that promoter hypermethylation is an important cause of the low expression of *ARID1A* in CRC cell lines (23) However, to the best of our knowledge, the role of histone deacetylation in *ARID1A* suppression in CRC has not yet been evaluated. For this reason, the current study aimed to investigate whether blocking histone deacetylation in CRC cell lines by TSA treatment affects the expression of *ARID1A* genes. 

## Methods

**Cell cultures**

Six CRC cell lines, LS180, SW480, SW742, SW48, HCT116, and HT-29/19, were investigated in this study. Cells were grown in DMEM (LS180) or RPMI 1640 (SW480, SW742, SW48, HCT116, and HT-29/19), supplemented with 1% penicillin/streptomycin, 10% fetal bovine serum (Gibco-BRL), and 2 mM glutamine, and then incubated in a 37 °C humidified atmosphere with 5% CO2.

**TSA treatment**

Cells were plated in T-25 flasks and allowed to attach for 24 h, after which cells were incubated for 24 h in media containing 300 nM TSA (Sigma-Aldrich, St. Louis, MO). Then, RNA was isolated as described below. DMSO (Sigma-Aldrich) was used as vehicle control. 

**RNA extraction and real-time quantitative PCR**

Total RNA was purified from CRC cell lines using Accuzol total RNA extraction reagent (Bioneer, Korea), according to the manufacturer’s instructions. Purified RNA quantification and purity was analyzed by a NanoDrop ND-1000 spectrophotometer (NanoDrop Technologies). The purified RNA integrity was detected by 0.8% agarose gel electrophoresis visualized by Gel-Red staining. Then, two micrograms of purified RNA were applied for cDNA synthesis by RevertAid First Strand cDNA Synthesis Kit (Fermentas), according to the manufacturer’s instructions.

**Figure 1:**
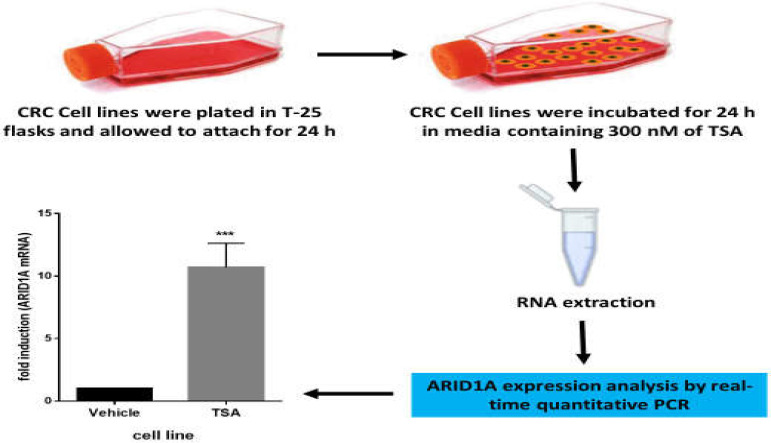
The schematic workflow for investigating the involvement of histone deacetylation in the ARID1A mRNA expression in CRC cell lines

The mRNA analysis of *ARID1A* and *GAPDH* as an internal housekeeping gene was determined by real-time quantitative PCR (qPCR) using SYBR Green master mix (ABI, UK) on an ABI 7500 Sequence Detection System (Applied Biosystems, USA). Primer sets used for qPCR assay were as follows: *ARID1A* (forward, 5’-CAGTACCTGCCTCGCACATA-3’ reverse, 5’ GCCAGGAGACCAGACTTGAG- 3’); *GAPDH* (forward, 5’- CGACCACTTTGTCAAGCTCA- 3’ reverse, 5’- AGGGGTCTACATGGCAACTG-3’). Next, qPCR reactions were performed in triplicate with a 60 °C annealing temperature and a total cycle number of 40. The relative expression of *ARID1A* mRNA was normalized to *GAPDH* mRNA levels and determined using formula 2^-ΔΔCT^. The workflow for investigating the role of histone deacetylation in reduced *ARID1A* expression in colorectal cancer cell lines is shown schematically in Figure 1.

**Statistical analysis**

Statistical analysis was done using SPSS software (Version 23) and GraphPad Prism (Version 6.01). Data reported as mean ± standard deviation (SD) was analyzed using one-way ANOVA, followed by Tukey’s multiple comparison tests. Differences with a *p*-value ≤ 0.05 were considered statistically significant. 

## Results

***ARID1A*** **expression is heterogeneous in CRC cell lines**

**Figure 2:**
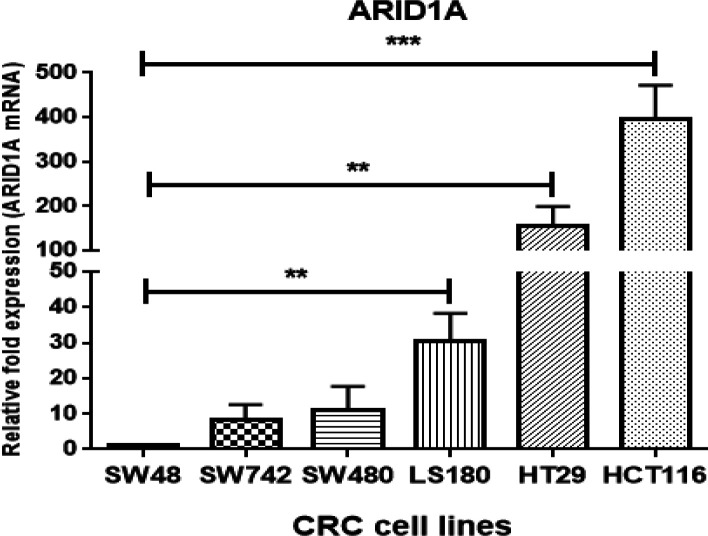
ARID1A relative mRNA expression in LS180, SW480, SW742, SW48, HCT116 and HT-29/19 cell lines measured by real-time quantitative PCR. -SW48 cell line with the lowest expression level of *ARID1A* -was used as a calibrator (expression level set to 1.0) and expressions in all other cell lines were presented as a fold-change relative to the SW48 cell line. *GAPDH* was used to normalize the *ARID1A* gene's expression values. Mean ± SD of three experiments is reported. (**p < 0.01, ***p < 0.001). Error bars show Standard Deviation (SD) of the mean for each triplicate experiment

We studied the expression profile of *ARID1A* by real-time quantitative PCR in six CRC cell lines, namely LS180, SW480, SW742, SW48, HCT116, and HT-29/19. *ARID1A* expression (normalized to *GAPDH* mRNA) was highly heterogeneous among cell lines (Figure 2). The lowest expression level of *ARID1A* was observed in SW48 cells, which were considered as a calibrator cell line for *ARID1A* expression (set at 1.0). The mRNA levels of *ARID1A* were significantly higher in HCT116, HT29, and LS180 cells compared to other cell lines (396.6-, 153.8-, and 30.5-fold more than SW48 cells, respectively). *ARID1A* mRNA expression was low in SW742 and SW480 cell lines with no significant difference with SW48 cells.

The SW48 cell line with the lowest expression level of *ARID1A* was used as a calibrator (expression level set to 1.0), and expressions in all other cell lines were presented as a fold-change relative to the SW48 cell line. *GAPDH* was used to normalize *ARID1A* gene expression values. Mean ± SD of three experiments is reported. (**p < 0.01, ***p < 0.001). Error bars show standard deviation (SD) of the mean for each triplicate experiment. 

**Figure 3:**
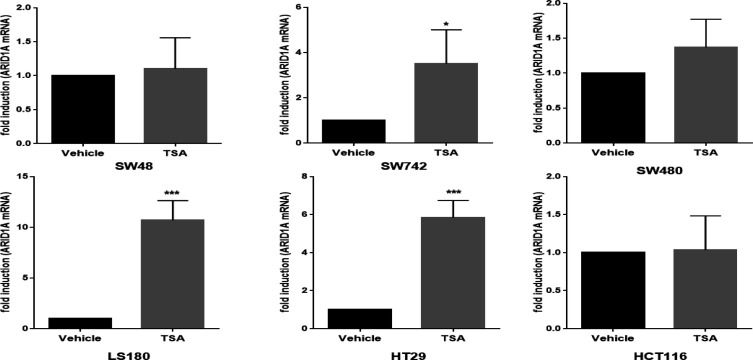
Treatment with trichostatin A (TSA) induces the mRNA expression of ARID1A in a cell line-dependent manner. mRNA relative expression of ARID1A after TSA treatment were examined by real-time quantitative PCR in CRC cell lines. ARID1A mRNA expression was normalized to GAPDH mRNA. The expression level of ARID1A in vehicle control (DMSO-treated cell lines) was set to 1 and the expression in each TSA-treated cell line was calculated as an n-fold difference relative to the vehicle control. Mean ± SD of three experiments is reported. (*p < 0.05, **p < 0.01, ***p < 0.001).

**Effects of TSA on the expression of** ***ARID1A*** **mRNA** **in human CRC cell lines**

To investigate the involvement of histone deacetylation in *ARID1A* mRNA expression, CRC cells were treated for 24 h with TSA, an inhibitor of histone deacetylase, and *ARID1A* expression was determined by real-time quantitative PCR. Treatment of SW48, HCT116, and SW480 cells with TSA had no significant effect on *ARID1A* expression (Figure 3); however, TSA treatment of SW742, HT29, and LS180 cells increased *ARID1A* expression 3.5-, 5.8- and 10.7-fold, respectively (Figure 3). 

## Discussion

Expression reduction of *ARID1A* and its tumor suppressor activity have been extensively described in a broad spectrum of cancers, including CRC. Previously, we reported decreased *ARID1A* expression in CRC cell lines (23). We and others have observed reduced *ARID1A* expression in CRC tumors (11, 18, 19, 23, 24, 30, 31). 

It has also been shown that the biallelic (*ARID1A*−/−) deletion of *ARID1A* alone led to the formation of invasive colorectal adenocarcinomas in mice (5). Therefore, exploring the molecular basis of this downregulation could lead to the development of ways for treating *ARID1A*-deficient colorectal cancer.

Interestingly, accumulating evidence indicates that epigenetic modifications such as DNA hypermethylation and histone modifications play significant roles in *ARID1A* gene downregulation in cancer (21, 23, 32). We previously reported that DNA hypermethylation is strongly correlated with *ARID1A* downregulation in CRC cell lines (23). Furthermore, a study which focused on decreased *ARID1A* expression in breast cancer demonstrated that histone modification and promoter hypermethylation are the main causes of *ARID1A* gene expression loss (21). In addition, treatment of gastric cancer cell lines with the methyltransferase inhibitor 5-aza-2’-deoxycytidine increased *ARID1A* expression, which indicated the important role of DNA hypermethylation in *ARID1A* downregulation (32). However, no study has experimentally examined whether histone modification plays a significant role in alteration of gene expression in colorectal cancer. Indeed, in our recent study, *ARID1A* expression was not restored after 5-aza treatment in SW480 and LS180 cell lines, which indicated the possibility of another mechanism in *ARID1A* downregulation, like histone modifications in these cell lines (23). Histone acetylation is by far the most studied histone modification (33). Decreasing histone acetylation plays a significant role in chromatin condensation and transcriptional repression of various genes in cancer (25). Therefore, we applied TSA in the current study to determine whether *ARID1A* expression can be increased in CRC cell lines by blocking histone deacetylation. Treatment with TSA influenced *ARID1A* expression in a cell line dependent manner. The highest upregulation of the gene was observed in the LS180 and HT29 cell lines and was almost 10-fold and 6-fold change, respectively. No such significant change was observed in the SW480, HCT116, or SW48 cell lines. Treatment of SW742 cells with TSA resulted in an almost threefold upregulation of *ARID1A* mRNA levels.

The current results suggest that TSA inhibition of histone deacetylation leads to increased *ARID1A* expression in LS180, HT29, and SW742 cells; however, it is still unknown whether this expression increase is due to a direct effect of TSA on the acetylation state of *ARID1A* genes. There are alternative explanations, e.g., TSA may alter the epigenome of genes that regulate *ARID1A* transcription, such as a coactivator. Therefore, more experimental studies that examine acetylation levels of histones within regulatory regions of the *ARID1A* gene after TSA treatment are necessary.

Clinical efficacy against cancer has been established for HDACs inhibitors, which can be considered as attractive targets for CRC therapy (26, 29). Upregulation of tumor suppressor gene *ARID1A *in CRC cell lines by HDACs pharmaceutical inhibitors seems to be a therapeutic approach for the treatment of *ARID1A*-deficient colorectal cancer.

Evaluation of cancer cells only, and not normal cells, can be considered as a limitation of this study. Evaluation of *ARID1A* expression in mRNA levels only, but not protein level, is another limitation of this study, which can be considered in future studies. Moreover, additional studies such as analysis of the *ARID1A* gene promoter histone modification levels by ChIP- qPCR (34) will be required to further elucidate the possible significance of *ARID1A* promoter histone deacetylation in *ARID1A* gene expression loss in CRC. In addition, the correlation between *ARID1A* expression and promoter histone modification needs to be assessed in patients with CRC. TSA also affects other epigenetic processes, including the methylation of DNA and histones (35, 36). Hence, further experiments will be required to definitely establish the molecular mechanism involved in *ARID1A* expression restoration by TSA.

Although the precise mechanisms accounting for decreased *ARID1A* expression in CRC are unknown, according to our cell line-based study, it is likely that epigenetic modifications play a role in *ARID1A* downregulation. More research is needed to support these findings.

## Conflict of interests

The authors declare that they have no conflict of interest.
